# Dopant Molecularization in β‑Ga_2_O_3_: Formation of N_2_ under Nonequilibrium Conditions

**DOI:** 10.1021/acs.jpclett.6c01536

**Published:** 2026-07-14

**Authors:** Iraida N. Demchenko, Asiyeh Shokri, Yevgen Syryanyy, Yevgen Melikhov, Maryna Chernyshova, Marcin Turek, Andrzej Droździel, Frans Munnik, Rafal Jakieła, Roman Minikayev, Jaroslaw Z. Domagala, Anastasiya Derkachova, Marcin Zaja̧c, Jan Krajczewski, Ewa Grzanka, Zbigniew Galazka

**Affiliations:** † The Centre for Advanced Materials and Technologies, CEZAMAT at the Warsaw University of Technology, 19 Poleczki St., Warsaw 02-822, Poland; ‡ 226287 Institute of Plasma Physics and Laser Microfusion, ul. Hery 23, 01-497 Warsaw, Poland; § Institute of Microelectronics and Optoelectronics, Warsaw University of Technology, ul. Koszykowa 75, 00-662 Warsaw, Poland; ⊥ Institute of Fundamental Technological Research, Polish Academy of Sciences, ul. Pawinskiego 5b, 02-106 Warsaw, Poland; & National Center for Nuclear Research, Andrzeja Sołtana 7, 05-400 Otwock, Poland; # Institute of Physics, Maria Curie-Sklodowska University, pl. M. Curie-Skłodowskiej 1, 20-031 Lublin, Poland; ★ Helmholtz-Zentrum Dresden-Rossendorf, Bautzner Landstraße 400, 01328 Dresden, Germany; ○ Institute of Physics PAS, al. Lotników 32/46, 02-668 Warsaw, Poland; △ National Synchrotron Radiation Centre SOLARIS, Jagiellonian University, Czerwone Maki 98, 30-392 Kraków, Poland; □ Faculty of Chemistry, University of Warsaw, Pasteura 1, 02-093 Warsaw, Poland; @ Institute of High Pressure Physics, Polish Academy of Sciences, ul. Sokolowska 29/37, 01-142 Warsaw, Poland; ∞ 28399 Leibniz-Institut für Kristallzüchtung, Max-Born-Straße 2 12489 Berlin, Germany

## Abstract

The microscopic fate of dopants introduced under nonequilibrium conditions remains largely unresolved in wide-band gap oxides. Using temperature-dependent N K-edge X-ray absorption spectroscopy, we directly resolve the local bonding configuration of implanted nitrogen in (100) β-Ga_2_O_3_. The spectra are dominated by a sharp π* resonance characteristic of N≡N bonding that systematically intensifies upon annealing, providing a direct spectroscopic fingerprint of molecular nitrogen formation. First-principles calculations and multiple-scattering simulations reproduce these spectral features and identify molecular N_2_ as the dominant dopant state. Rather than forming substitutional acceptors, implanted nitrogen evolves toward N_2_-like configurations stabilized in defect-rich environments associated with local β→γ-like structural motifs. This behavior reflects a thermally driven reconfiguration of nitrogen within the damaged layer. These results demonstrate that dopant incorporation can proceed via molecularization pathways that bypass conventional substitutional doping, providing a general mechanism for dopant deactivation under nonequilibrium incorporation conditions in oxides.

## Graphical abstract

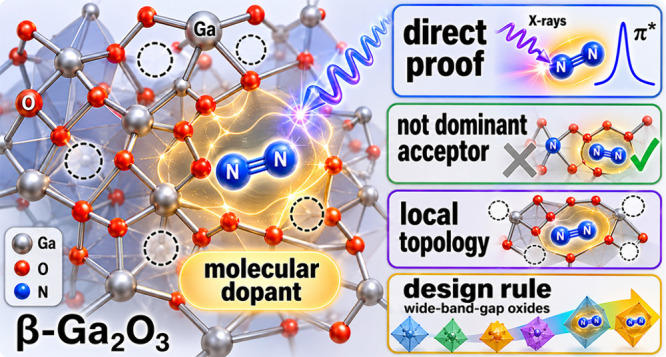

β-Ga_2_O_3_ has emerged as a prototypical ultrawide band gap (WBG) semiconductor for next-generation high-power electronics owing to its large breakdown field, availability of bulk substrates, and compatibility with scalable growth technologies.^1–4^ Despite rapid advances in device architectures, a fundamental materials limitation persists: the absence of reliable and stable p-type conductivity.^5,6^ The inability to realize effective acceptor doping in β-Ga_2_O_3_ constrains the development of bipolar and complementary device concepts and remains one of the central unresolved problems in this material system. Importantly, this limitation reflects not only energetic constraints but also the fundamentally unknown chemical form in which dopants, in this case nitrogen, incorporate under nonequilibrium conditions. A key unresolved question is whether dopant atoms introduced under strongly nonequilibrium conditions remain isolated within the lattice or instead undergo spontaneous pairing or molecularization, thereby suppressing their intended electronic activity. In such regimes, dopants cannot be assumed to behave as isolated atomic defects but may instead form chemically distinct species with different bonding and electronic structure. More generally, it remains unclear whether dopants introduced far from equilibrium remain as isolated atomic defects or transform into molecular species with fundamentally different electronic behavior, representing a distinct mode of dopant incorporation.

Nitrogen in β-Ga_2_O_3_ provides a particularly intriguing case. Despite being a long-considered acceptor candidate due to its chemical similarity to oxygen and its role in other oxide and nitride semiconductors, it has repeatedly failed to produce hole conductivity. First-principles calculations predict that substitutional nitrogen on oxygen sites (*N*_*O*_) forms deep acceptor states,^7^ while various nitrogen-related complexes may act as compensating centers or become energetically competitive depending on Fermi-level position and growth conditions.^1,6^ In particular, theoretical studies indicate that nitrogen can exhibit amphoteric behavior and form multiple configurations, including complexes with oxygen vacancies, which may substantially modify its electronic activity.^2,6–8^ This configurational flexibility makes nitrogen a sensitive probe of competing atomic and molecular incorporation pathways.

Experimentally, nitrogen incorporation, whether during growth, plasma exposure, or ion implantation, has consistently resulted in semi-insulating behavior and deep defect levels rather than shallow acceptor activation.^3,9,10^ Electrical measurements reveal strong compensation and trap formation, yet the microscopic origin of this self-compensation remains unresolved. In particular, the lack of direct spectroscopic identification of the chemical state of nitrogen represents a critical gap in understanding its electronic inactivity. Direct experimental determination of the local bonding configuration of nitrogen in β-Ga_2_O_3_, therefore, remains scarce and largely indirect.

Evidence from other wide-band gap oxides suggests that the chemical state of nitrogen may deviate significantly from the simple substitutional acceptor picture. In ZnO, N K-edge X-ray absorption near edge structure (XANES) studies have demonstrated that nitrogen frequently forms molecular N_2_-like configurations within the lattice rather than isolated substitutional *N*_*O*_ centers, even when nominally introduced as an acceptor dopant.^11,12^ The presence of characteristic π* resonances associated with N–N bonding provided direct spectroscopic evidence for such molecular configurations. Moreover, using electron energy loss spectroscopy, it was shown^13^ that after annealing, zinc vacancy clusters (V_Zn_) filled with N_2_ are formed, interpreted as evidence that nitrogen does not stabilize in the substitutional *N*_*O*_ configuration and thereby limits p-type doping. These findings highlight that identical nominal dopant concentrations can correspond to fundamentally different local bonding environments with drastically different electronic consequences. The latter suggests that molecular dopant formation may be a general phenomenon in wide-band gap oxides rather than a material-specific anomaly.

For β-Ga_2_O_3_, N K-edge XANES investigations similarly indicate the coexistence of distinct nitrogen species depending on processing conditions. Experimental spectra have revealed features consistent with both Ga–N bonded states and molecular-like nitrogen configurations, with the relative contribution evolving under thermal treatment and nitridation conditions.^14^ These observations suggest that nitrogen incorporation does not necessarily yield a unique structural motif and may instead reflect a competition between atomic and molecular forms of the dopant, leading to the fact that metastable or molecular forms may be stabilized under quasi-equilibrium or defect-rich environments.

The atomic configuration of nitrogen depends critically on the incorporation pathway. While growth and high-temperature nitridation may approach near-equilibrium bonding environments, ion implantation represents a strongly nonequilibrium process, generating a dense population of vacancies, interstitials and defect complexes. Under these conditions, the concept of a well-defined substitutional dopant becomes insufficient to describe impurity incorporation. This defect-rich environment fundamentally reshapes the thermodynamic landscape, potentially stabilizing nitrogen configurations distinct from isolated substitutional acceptors, including vacancy-assisted complexes or N_2_-like species embedded in the lattice.^2^ Consequently, the microscopic mechanism governing nitrogen self-compensation under implantation conditions remains experimentally unresolved. Specifically, it remains unclear whether implanted nitrogen predominantly occupies substitutional oxygen sites, forms vacancy-associated complexes, or evolves toward molecular N_2_-like configurations during postimplantation thermal processing. Here we directly probe the local configuration of implanted nitrogen using temperature-dependent N K-edge XANES, aiming to resolve the microscopic bonding environment responsible for nitrogen-induced compensation in β-Ga_2_O_3_. Resolving this question requires a direct, element-specific probe of the local bonding configuration. In this work, we test the hypothesis that dopant molecularization represents a dominant and previously overlooked incorporation pathway under implantation conditions in Ga_2_O_3_.

Figure 1(a) presents the central experimental observation. All N-implanted β-Ga_2_O_3_ samples exhibit a pronounced near-edge resonance at the N K-edge with a line shape dominated by a strong π* feature characteristic of N–N bonding. The spectra are clearly distinct from crystalline GaN (orange line), demonstrating that the implanted nitrogen does not predominantly form an extended Ga–N network. Instead, the spectral response directly indicates that nitrogen predominantly exists in a molecular form rather than as a network-forming dopant species.

**Figure 1:**
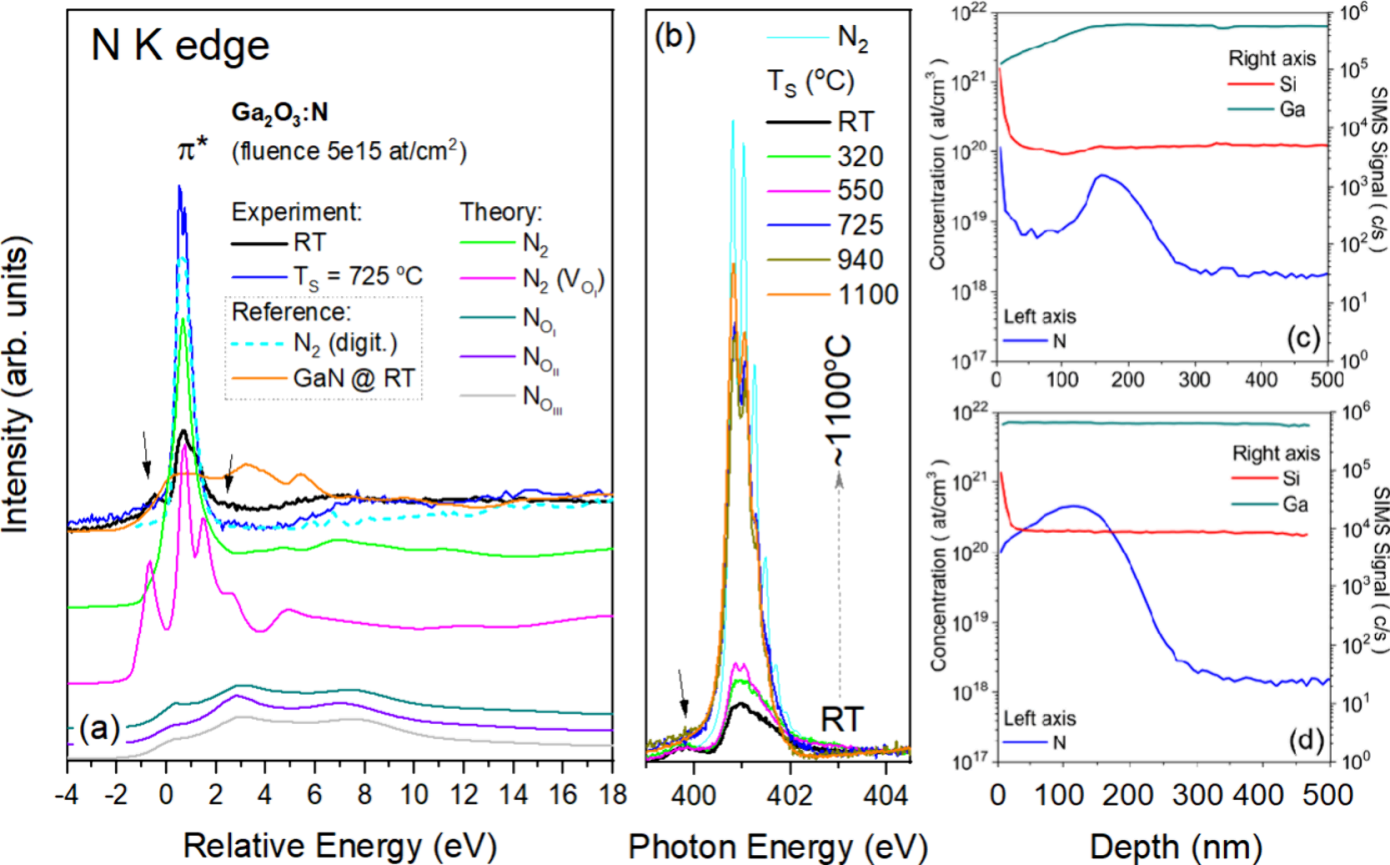
(a) Normalized N K-edge XANES spectra of β-Ga_2_O_3_:N with fluence 5 × 10^15^ atoms/cm^2^ implanted at room temperature (RT) and selected annealing (*T*_S_) at 725 °C measured using FLUO detection mode. The black/red/blue/cyan solid lines combined at the top represent the experimental spectra. Spectrum corresponding to molecular nitrogen was digitized from ref 15. Theoretical spectra calculated using FDMNES software were spread for clarity. Models correspond to three different substitutional *N*_*O*_(combined in down position), molecular nitrogen formed from two interstitial N (i5–i9, notation from ref 7), and a similar molecular model in *V*_*O*_. (b) high-resolution XANES spectra for implanted specimens in the whole range of annealing temperatures compared to the molecular nitrogen spectrum. SIMS depth profiles of the investigated samples (c) implanted at RT and (d) subsequently annealed at 1100 °C. The nitrogen distribution maximum in the sample implanted at RT is in good agreement with SRIM calculations shown in Figure 3(b).

With increasing annealing temperature, the π* resonance systematically sharpens and gains spectral weight, while the overall near-edge profile evolves toward a more symmetric molecular-like response (Figure 1(b)). This evolution reflects a progressive reorganization of the local nitrogen environment rather than a simple redistribution among defect configurations. The most pronounced spectral changes occur between 550 and 725 °C, a temperature range associated with substantial defect recovery and enhanced defect mobility in implanted β-Ga_2_O_3_.^16^ Under these conditions, isolated nitrogen species can undergo short-range migration and aggregation into energetically favorable N–N bonded configurations.^6^ Consistently, the temperature dependence reveals a thermally activated conversion of nitrogen toward molecular N_2_ species rather than activation into substitutional acceptors.

An additional spectral signature supporting this interpretation is the dip visible around ∼4 eV above the edge. This dependence is absent in the spectrum of the room-temperature (RT) sample but develops after annealing at 725 °C (blue line). At this temperature the experimental spectrum closely resembles the reference spectrum of molecular N_2_ (cyan line). The simultaneous presence of the sharp π* resonance and the characteristic postedge dip therefore provides strong spectroscopic evidence for the formation of molecular nitrogen species within the implanted Ga_2_O_3_ layer.

To identify microscopic configurations compatible with the observed spectral line shape, the experimental spectra were compared with first-principles-based XANES simulations for several candidate nitrogen configurations, see Figure 1(a). The tested models included: (i) substitutional nitrogen on oxygen sites (*N*_*O*_, three curves combined in the lower panel), (ii) interstitial N–N paired configurations (i5–i9), representing positions adopted from ref 7, magenta line, (iii) vacancy-assisted molecular configurations (i5–i9–*V*_*O*_, green line). The substitutional *N*_*O*_ models fail to reproduce the dominant π* resonance and do not capture the experimental near-edge line shape. In contrast, molecular configurations reproduce both the intense π* resonance and the characteristic pre-π* spectral shoulder. Within experimental sensitivity, the data therefore constrain the dominant nitrogen state in the implanted layer to N_2_-like configurations, although a minor contribution from nonmolecular motifs cannot be excluded within the intermediate thermal window. Nevertheless, the dominance of the π* resonance unambiguously identifies molecular N_2_ as the primary chemical state of nitrogen.

The combination of temperature-dependent N K-edge XANES and first-principles modeling reveals that nitrogen introduced by ion implantation in β-Ga_2_O_3_ (100) does not predominantly incorporate as substitutional *N*_*O*_ defects but instead self-organizes into N–N bonded molecular configurations stabilized by implantation-induced disorder. While substitutional nitrogen has long been considered the most likely configuration for nitrogen doping in gallium oxide,^6,7^ direct spectroscopic verification of this assumption has remained limited, but our spectroscopic results indicate that molecular nitrogen plays the dominant role in the implanted and annealed gallium oxide. This establishes molecular nitrogen as the relevant dopant state controlling the electronic behavior of the system.

A key insight emerges from the structural relaxation of the candidate defect configurations. In the isolated N_2_ model calculation the optimized N–N bond length is 1.094 Å, in excellent agreement with the experimental bond length of the free nitrogen molecule (∼1.10 Å). This confirms that the computational framework accurately reproduces the intrinsic molecular geometry of nitrogen. When the N–N pair is embedded in the oxide sublattice (our case oxygen vacancy), however, the bond length increases to 1.165 Å. Such elongation is characteristic of partial occupation of antibonding π* orbitals and indicates that the molecule is weakly charged through interaction with the surrounding lattice. In molecular terms, the configuration can therefore be described as a partially reduced species *N*_2_^δ−^ stabilized by the defect environment representing a chemically distinct dopant state stabilized by charge transfer from the oxide lattice. This interpretation is further supported by literature showing that electron transfer/back-donation into antibonding orbitals weakens the N–N bond, both in oxide/matrix hosts and in electronically activated dinitrogen complexes.^12,13,17,18^

The Bader charge analysis provides direct insight into the electronic origin of this bond elongation. For substitutional nitrogen on the oxygen site (*N*_*O*_), the calculated charge corresponds to approximately −1*e* on the nitrogen atom. This reflects the ionic character of the Ga–N bond and is consistent with the expected electronic configuration of substitutional nitrogen in an oxide lattice. In contrast, the interstitial N–N configuration (i5–i9) yields nearly neutral nitrogen atoms, with the total charge of the N_2_ unit remaining close to zero. This behavior is typical of a covalently bonded molecular unit embedded in the lattice rather than two independent atomic defects. This distinction directly separates molecular dopant states from conventional atomic defect descriptions. A different situation emerges when an oxygen vacancy is present in the vicinity of the nitrogen pair. In the i5–i9–*V*_*O*_ configuration both nitrogen atoms acquire additional electronic density, leading to partial negative charging of the molecular unit. Oxygen vacancies in oxides are well-known to act as electron donors, and the calculated Bader charges confirm that a fraction of this donated charge populates the antibonding orbitals of the nitrogen molecule. The resulting charge state weakens the N–N bond and naturally explains the calculated bond elongation relative to the neutral molecule. The simultaneous increase in bond length and accumulation of electronic density on the molecular unit therefore provides a consistent structural and electronic fingerprint of vacancy-stabilized molecular nitrogen providing a clear electronic signature of molecular dopant formation.

The molecular character of the N–N complexes is further reflected in the calculated charge-transition levels shown in Figure S1 in Supporting Information. For the interstitial pair configuration (i5–i9), the calculated transitions occur at approximately E_V_+0.68 and E_V_+1.63 eV, while the vacancy-assisted configuration (i5–i9–V_O_) exhibits transitions at E_V_+0.21 and E_V_+3.78 eV. These levels span a broad portion of the band gap and demonstrate that the N–N complex can stabilize several charge states. Such behavior is characteristic of molecular defects whose electronic structure is governed by the filling of bonding and antibonding orbitals associated with the N–N unit. For comparison, hybrid Density Functional Theory (hybrid-DFT) calculations performed for substitutional *N*_*O*_ in β-Ga_2_O_3_ reveal a distinctly different electronic behavior. The calculated thermodynamic transition levels form a compact sequence close to the valence-band edge. In particular, the (+2/+1) and (+1/0) transitions occur within approximately 0.6–1.6 eV above the valence band maximum (VBM), while the (0/–1) transition lies deeper in the gap at roughly 2–3.6 eV depending on the specific oxygen site. Such a hierarchy of charge states is characteristic of a localized atomic acceptor whose electronic structure is primarily governed by N 2p states hybridized with the valence band of the oxide lattice. In contrast, the N–N configurations considered in the present work exhibit thermodynamically accessible charge states distributed over a much broader energy range across the band gap (we will come back to this below). This behavior is naturally expected for molecular complexes, where the electronic structure is controlled by the occupation of molecular orbitals associated with the N≡N bond rather than by a single impurity level. This fundamental difference highlights that molecular dopants cannot be described within the conventional framework of isolated defect levels.

Importantly, the structural analysis demonstrates that the formation of molecular nitrogen does not require the presence of a stable γ-Ga_2_O_3_ phase. X-ray diffraction (XRD) patterns (Figure 2) show that implantation initially induces a metastable γ component in the damaged region, while annealing restores long-range β-phase order but does not completely recover the shape of the virgin β-Ga_2_O_3_ reflection, in particular, residual broadening/asymmetry remains on the low-angle side of the main peak. The important point is therefore not complete structural recovery, but the different response of the two structural components to annealing. The γ-like long-range diffraction feature is strongly reduced or disappears after annealing, whereas residual implantation-induced strain, defect-related lattice distortion, mosaicity, or local disorder may persist in the β-Ga_2_O_3_ matrix. Moreover, the persistence and strengthening of the molecular spectral signature even after the disappearance of the γ diffraction features indicate that nitrogen molecularization is governed by local defect environments rather than by long-range polymorphic stability. This demonstrates that molecularization is controlled by local atomic topology rather than crystallographic phase identity. In this sense, the implantation-induced defect landscape provides the structural conditions necessary for stabilizing N–N bonded units within the oxide lattice. A comparison of the β and γ crystal structures provides an additional structural perspective on this stabilization mechanism. Several lattice positions characteristic of the γ polymorph correspond closely to interstitial sites within the β-Ga_2_O_3_ framework.^19^ Ion implantation therefore creates a defect landscape in which local atomic arrangements temporarily reproduce γ-like coordination motifs inside the β lattice effectively creating transient structural motifs that promote N–N bond formation. This observation indicates that the stabilization of N_2_ units is governed primarily by local defect topology rather than by the long-range crystallographic identity of the host phase. Even after the γ diffraction signatures disappear, implantation-induced vacancy clusters and distorted coordination motifs can remain locally preserved and continue to act as trapping environments for molecular nitrogen.

**Figure 2:**
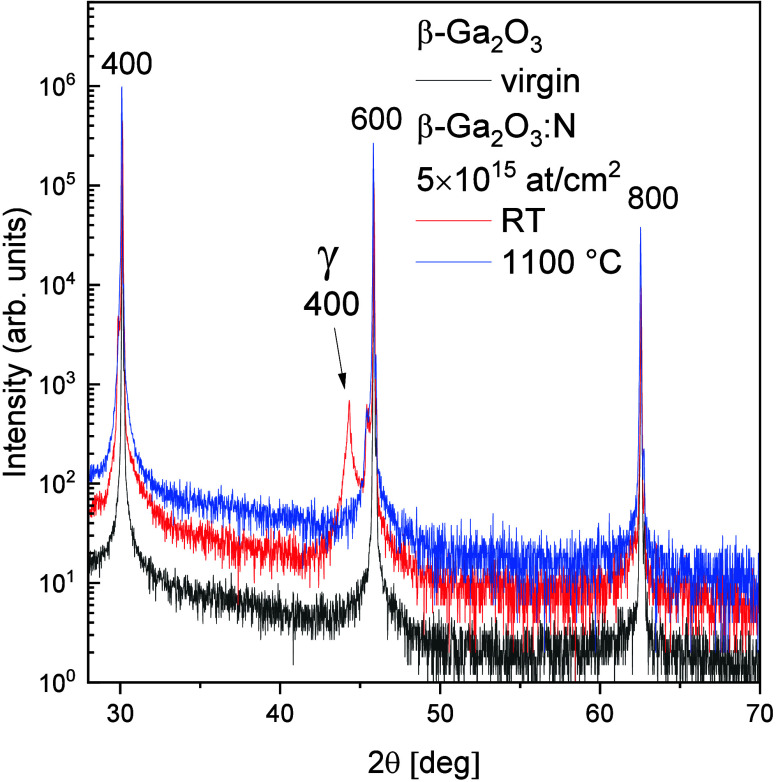
Wide-angle XRD profiles of N implanted β-Ga_2_O_3_ with fluence 5 × 10^15^ at/cm^2^ at different annealing temperatures and virgin monocrystal shown for comparison. Note that additional diffraction peak corresponding to the γ-phase, indicated by arrow, for the RT implantation protocol is revealed. In the implanted sample after annealing at 1100 °C, the γ-phase signal is not detectable.

Within such environments, enlarged interstitial volumes and under-coordinated motifs can trap nitrogen atoms. When two nitrogen atoms occupy adjacent positions within these motifs, the formation of an N–N bond becomes energetically favorable (see Figure S1 in the Supporting Information), leading to the stabilization of molecular N_2_-like complexes within the β-Ga_2_O_3_ matrix. Even after annealing restores long-range β-phase order, remnants of these local defect topologies may persist and act as metastable trapping sites for molecular nitrogen. Raman measurements independently confirm the presence of molecular nitrogen in the samples (see Figure S2 in the Supporting Information). Rutherford backscattering spectrometry in channeling mode (RBS/c) results (Figure 3) show that, after annealing, the defect-related signal remains confined to the near-surface implanted region, although its profile deviates from the as-implanted stopping and range of ions in matter (SRIM) distribution, indicating thermally driven defect redistribution. While outward nitrogen migration cannot be excluded based on RBS/c alone, which cannot detect nitrogen directly, the “Relative Defect Concentration” reflects defect accumulation rather than nitrogen content and therefore does not provide direct evidence of nitrogen diffusion. In contrast, the SRIM-predicted nitrogen profile for RT implantation is in very good agreement with the secondary-ion mass spectrometry (SIMS) data (Figure 1(c)). Crucially, the SIMS comparison before and after annealing (Figure 1(d)) reveals a clear shift of nitrogen toward the surface, providing direct evidence of diffusion toward the near-surface region upon thermal treatment consistent with the redistribution of molecular nitrogen within the defect-rich layer.

**Figure 3:**
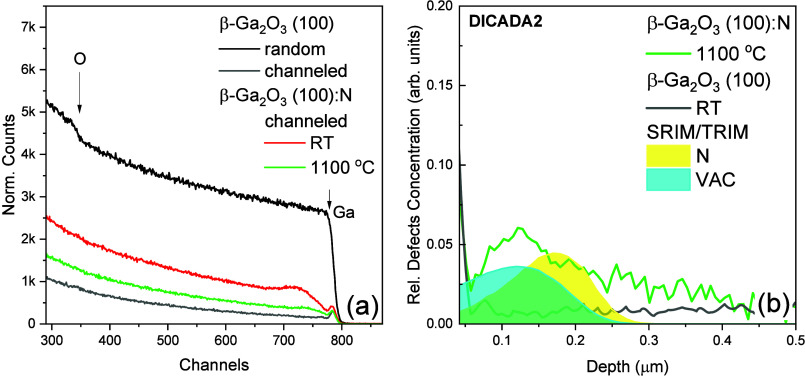
(a) RBS random and [201] aligned (channeled) spectra for the β-Ga_2_O_3_ monocrystals implanted by N at different substrate temperatures (*T*_S_). (b) Relative defect concentration (RDC) as a function of depth obtained using the DICADA program^20^ from the RBS spectra of the virgin sample measured at RT and the implanted sample after annealing at 1100 °C. For comparison, the N (yellow) and Ga/O vacancy (cyan) profiles calculated using the TRIM/SRIM program^21^ are also shown (unscaled).

It should be noted that first-principles calculations for equilibrium defect formation in β-Ga_2_O_3_ predict relatively high formation energies for isolated interstitial N_2_ species. However, ion implantation represents a strongly nonequilibrium process that generates a high density of vacancies, interstitials, and defect clusters. Such defect-rich environments can substantially modify the local thermodynamic landscape and provide metastable trapping sites where molecular nitrogen configurations become energetically stabilized even when such configurations are not favored under equilibrium conditions. Additional support for a molecular interpretation of nitrogen-related defects comes from electrical defect spectroscopy studies of nitrogen-implanted β-Ga_2_O_3_. Deep-level optical spectroscopy has revealed a prominent nitrogen-related trap located approximately *E*_C_ – 2.9 eV below the conduction band. Remarkably, this defect is characterized by an unusually large Franck–Condon relaxation energy of about 1.4 eV, indicating extremely strong lattice relaxation accompanying the electronic transition.^1,22^ Such a large Franck–Condon shift is exceptional even among WBG semiconductors and implies a defect configuration undergoing substantial structural reorganization during charge capture or emission. Molecular defect complexes provide a natural microscopic explanation for this behavior. Changes in the charge state of an N–N unit modify the occupation of antibonding π* orbitals and directly alter the N–N bond length, thereby inducing significant relaxation of the surrounding lattice. This strong coupling between electronic occupation and molecular geometry offers a natural explanation for the unusually large relaxation energy reported for nitrogen-related trap states and further supports the molecular nature of the underlying defect states.

Taken together, the structural, electronic, and spectroscopic data suggest a heterogeneous nitrogen speciation during the thermal evolution of the implanted layer. At lower temperatures, the system likely contains a heterogeneous mixture of configurations, including substitutional *N*_*O*_ defects predicted by first-principles calculations as deep acceptors, interstitial N–N pairs (i5–i9), and vacancy-assisted molecular complexes (i5–i9–*V*_*O*_). The coexistence of these configurations is consistent with the strongly nonequilibrium nature of ion implantation and the broad distribution of defect environments created in the damaged layer, where both atomic and molecular configurations compete during thermal evolution. As the material is annealed, defect recombination and structural relaxation progressively favor the stabilization of molecular nitrogen species. Consequently, after annealing at temperatures approaching 725 °C, the nitrogen population becomes dominated by N_2_-like configurations trapped within the oxide matrix, indicating a thermally driven transition from atomic to molecular dopant states.

This mechanism provides a natural explanation for the well-known difficulty of achieving effective p-type doping in gallium oxide under ion implantation conditions. In this framework, nitrogen introduced via implantation does not behave as a simple electrically inactive impurity; instead, it self-organizes into molecular-like complexes that are electronically decoupled from the valence band, thereby preventing the formation of shallow acceptor states. In this way, the implantation-driven incorporation pathway effectively bypasses the conventional substitutional doping channel by transforming dopants into electronically decoupled molecular entities.

While first-principles calculations predict substitutional nitrogen at the oxygen site as the thermodynamic ground state, the conditions associated with ion implantation deviate strongly from equilibrium. The implantation process generates a dense population of defects and introduces nitrogen atoms in close spatial proximity within collision cascades. Under these conditions, nitrogen atoms can readily interact and form N–N bonded configurations. Once formed, such paired configurations represent locally stable states. Subsequent annealing is expected to promote both the formation and stabilization of these molecular species through defect recovery and enhanced nitrogen mobility, thereby increasing their relative population within the implanted layer. The present results establish dopant molecularization as an experimentally verified incorporation pathway in ion-implanted β-Ga_2_O_3_. Combined with analogous observations of molecular N_2_ formation in nitrogen-implanted ZnO, we hypothesize that dopant molecularization may represent a broader defect-assisted incorporation pathway in wide-band gap oxides subjected to nonequilibrium processing, rather than a phenomenon unique to β-Ga_2_O_3_.

In summary, the combination of temperature-dependent N K-edge XANES and first-principles modeling reveals that nitrogen introduced by ion implantation in (100) β-Ga_2_O_3_ does not predominantly incorporate as substitutional *N*_*O*_ defects but instead self-organizes into N–N bonded molecular configurations stabilized by implantation-induced disorder. The observed evolution is consistent with a defect-assisted molecularization process, in which increasing defect mobility during annealing promotes the aggregation of initially dispersed nitrogen species into energetically favorable N_2_-like configurations.

## Experimental Methods

Crystal samples were prepared from a 2-in.-diameter bulk β-Ga_2_O_3_ single crystal grown along the [010] crystallographic direction using the Czochralski method at the Leibniz-Institut für Kristallzüchtung (Berlin, Germany). The crystal was grown from an Ir crucible with an oxygen concentration of 8 vol % in the growth atmosphere with no intentional dopants (see^23,24^ for further details). The samples of size 5 mm × 5 mm × 0.5 mm were (100) oriented and a double-sided chemical-mechanical polishing was performed. The samples were semiconducting with the free electron concentration and electron mobility (from Hall effect measurements) of 3.4 × 10^17^ cm^–3^ and 118 cm^2^ V^–1^ s^–1^, respectively. Monocrystalline β-Ga_2_O_3_ samples were implanted with N ions of energy 100 keV at room temperature (RT). Base pressure in the irradiation chamber was about 10^–6^ mbar. Irradiation flux was of order 0.25 μA/cm^2^ and fluence was set to 5 × 10^15^ ions/cm^2^. A custom-made arc discharge ion source with an internal evaporator was used.^25,26^ The implantation angle was set at approximately 7° from the surface normal to avoid channeling.

To monitor lattice disorder and the nitrogen lattice-site distribution with depth resolution, Rutherford backscattering spectrometry in channeling mode (RBS/c) measurements were carried out using a 1.7 MeV He^+^ beam and a Si PIN diode detector positioned at a backscattering angle of 170°.

Concentrations of Si, Ga, and N were determined by secondary-ion mass spectrometry (SIMS) using a CAMECA IMS-6F ion microprobe.

X-ray absorption experiments were performed at the Solaris synchrotron on the PIRX beamline. All in-situ/ex-situ X-ray absorption near edge structure (XANES) measurements were performed at RT on samples subjected to stepwise annealing in the temperature range of 320–1100 °C. The XANES spectra were obtained by recording the total fluorescence yield (TFY or FLUO) signal from the samples while scanning the photon energy across the N K-edge region. The measurements were carried out with the polarization vector of the synchrotron radiation oriented close to the “magic” angle,^27^ i.e., the angle at which the absorption cross section becomes independent of orbital orientation. Annealing was carried out under ultrahigh vacuum conditions (pressure below 6 × 10^–9^ mbar) in a dedicated chamber. Depending on the heating mode, the heating rate varied between 3 and 10 °C·s^–1^, using either radiative heating alone or a combination of radiative and electron-beam heating. At each temperature step, the samples were annealed for approximately 10 min. At temperatures below 400 °C, the sample temperature was monitored using a calibrated thermocouple mounted on the manipulator. Above 400 °C, the temperature was monitored using an Optris CSvision R2ML pyrometer. After normalization to the photon flux, the recorded N K-edge XANES spectra were subjected to subtraction of a linear background fitted to the flat pre-edge region. For quantitative comparison, the spectra were subsequently normalized to the atomic limit in the region approximately 30 eV above the absorption edge, where angular dependence is negligible.

The X-ray diffraction (XRD) experiment was performed in a θ–2θ scan geometry using a laboratory diffractometer equipped with a Cu X-ray tube (Cu Kα_1_) and a Johansson monochromator.

First-principles density functional theory (DFT) calculations were performed using VASP (v. 6.3.2) that employs the projector-augmented wave method.^28–31^ The Heyd–Scuseria–Ernzerhof (HSE06) hybrid functional^32^ with the fraction of the Fock exchange α = 0.35, and a screening parameter μ = 0.20 Å^–1^ was used. The pristine β-Ga_2_O_3_ has a monoclinic crystal structure with space group *C*2/*m*, with the lattice constants *a* = 12.23 Å, *b* = 3.04 Å, and *c* = 5.80 Å.^7,33,34^ Defect formation energies and thermodynamic charge transition levels were calculated in a relaxed 160-atom 1 × 4 × 2 supercell using standard formalisms.^7,35–37^ Further simulation details are provided in the Supporting Information.

The N K-edge XANES spectra were calculated from the relaxed atomic structures using the FDMNES code.^38^ We primarily employed the computationally efficient multiple-scattering muffin-tin approximation (MTA), which proved sufficient for atoms occupying regular lattice sites, including the Ga K-edge and O K-edge of Ga_2_O_3_ and the N K-edge of Ga_2_O_3_:N. Full-potential finite-difference (FDM) calculations confirmed that MTA yields very similar spectra that accurately reproduce bulk undisturbed Ga_2_O_3_ experimental data (not shown).

## Supplementary Material



## Data Availability

The data that support the findings of this study are openly available in RepOD - Repository for Open Data at https://doi.org/10.18150/WEDASZ

## Notes

DFT computational details and auxiliary results together with formation energies of nitrogen-related defects in β-Ga_2_O_3_; Raman spectroscopy experimental details and results (PDF)
