# Supplementary material for: Dopant Molecularization in β‑Ga2O3: Formation of N2 under Nonequilibrium Conditions
Source: J Phys Chem Lett. 2026 Jul 14;17(29):8232–9. doi: 10.1021/acs.jpclett.6c01536 (PMC13403296; doi:10.1021/acs.jpclett.6c01536)
Supplement: Supplementary file 1 [file jz6c01536_si_001.pdf]

Supporting Information: Dopant Molecularization in β-Ga_2O_3: Formation of N_2 under Nonequilibrium Conditions

Iraida N. Demchenko, [1,2,*] *Asiyeh Shokri,* [2] *Yevgen Syryanyy,* [3] *Yevgen Melikhov,* [4] *Maryna Chernyshova* [5], *Marcin Turek,* [6] *Andrzej Droździel,* [6] *Frans Munnik,* [7] *Rafal Jakieła,* [8] *Roman Minikayev,* [8] *Jaroslaw Z. Domagala,* [8] *Anastasiya Derkachova,* [8] *Marcin Zając,* [9] *Jan Krajczewski,* [10] *Ewa Grzanka,* [11] *Zbigniew Galazka,* [12]

[1] The Centre for Advanced Materials and Technologies, CEZAMAT at the Warsaw University of Technology, 19 Poleczki St, Warsaw 02-822, Poland

[2] Institute of Plasma Physics and Laser Microfusion, ul. Hery 23, 01-497 Warsaw, Poland

[3] Institute of Microelectronics and Optoelectronics, Warsaw University of Technology, ul. Koszykowa 75, 00-662 Warsaw, Poland

[4] Institute of Fundamental Technological Research Polish Academy of Sciences, ul. Pawinskiego 5b, 02-106 Warsaw, Poland

[5] National Center for Nuclear Research, Andrzeja Sołtana 7, 05-400 Otwock, Poland

[6] Institute of Physics, Maria Curie-Sklodowska University, pl. M. Curie-Skłodowskiej 1, 20-031 Lublin, Poland

[7] Helmholtz-Zentrum Dresden-Rossendorf, Bautzner Landstraße 400, 01328 Dresden, Germany

[8] Institute of Physics PAS, al. Lotników 32/46, 02-668 Warsaw, Poland

[9] National Synchrotron Radiation Centre SOLARIS, Jagiellonian University, Czerwone Maki 98, 30-392 Kraków, Poland

[10] Faculty of Chemistry, University of Warsaw, Pasteura 1, 02-093 Warsaw, Poland

[11] Institute of High Pressure Physics Polish Academy of Sciences, ul. Sokolowska 29/37, 01-142 Warsaw, Poland

[12] Leibniz-Institut für Kristallzüchtung, Max-Born-Straße 2 12489 Berlin, Germany

*E-mail: iraida.demchenko@pw.edu.pl

S1. DFT Computational Details and Auxiliary Results

First-principles DFT simulations were performed using Vienna *Ab* initio Simulation Package (VASP, v. 6.3.2) that employs the projector-augmented wave method. [1-4] The spin-polarized hybrid DFT was chosen because it is an established approach for studying semiconducting materials that successfully predicts structural, electronic, and optical properties.

During the optimization of the pristine β-Ga_2O_3 structures comprising 20 atoms in the conventional unit cell, we chose an energy cutoff of 510 eV, force tolerance on each ion was set below 0.03 eV $Å^{-1}$, and tolerance of total energy was set to less than 10^{-4} eV per atom with 2 × 8 × 4 Γ-centered Monkhorst-Pack k-points for a unit cell.

For the structures with defects, a 1 × 4 × 2 supercell of 160 atoms was prepared, and a 1 × 1 × 1 Γ-centered Monkhorst–Pack was used.

The standard procedure to compute the formation energies was utilized as outlined in Ref. [5] with the Freysoldt-Neugebauer-Van de Walle correction term to allow the elimination of false electrostatic interactions between charged defect structures. [6]

The experimental value of the dielectric constant used to calculate the correction term E_{corr} was chosen to be 10. [7] The defects in charge states of +3, +2, +1, 0, -1, -2, and -3 were considered.

In addition to the formation energy curve for a chosen defect, the thermodynamic charge transition level can be identified using [5]

$$\varepsilon\left({q_1}/{q_2}\right) = \frac{E^f_{q_1}|_{E_{F=0}} - E^f_{q_2}|_{E_{F=0}}}{q_2 - q_1} \qquad (1)$$

where $E^f_q|_{E_{F=0}}$ is the formation energy of the structure with the defect in charge state q evaluated at EF = 0.

The following values for the chemical potentials of the elements were chosen (analogous to Ref. [8]):

μ_O = -11.075 eV (from O_2 molecule to be used for the Ga-rich condition),

μ_{Ga} = -3.38 eV (from bulk Ga metal to be used for the Ga-rich conditions),

μ_N = -10.78 eV (from N_2 molecule to be used for the Ga-poor condition),

μ_N = -12.09 eV (from GaN to be used for the Ga-rich condition),

ΔE = -12.23 eV (Formation Enthalpy of β-Ga_2O_3).

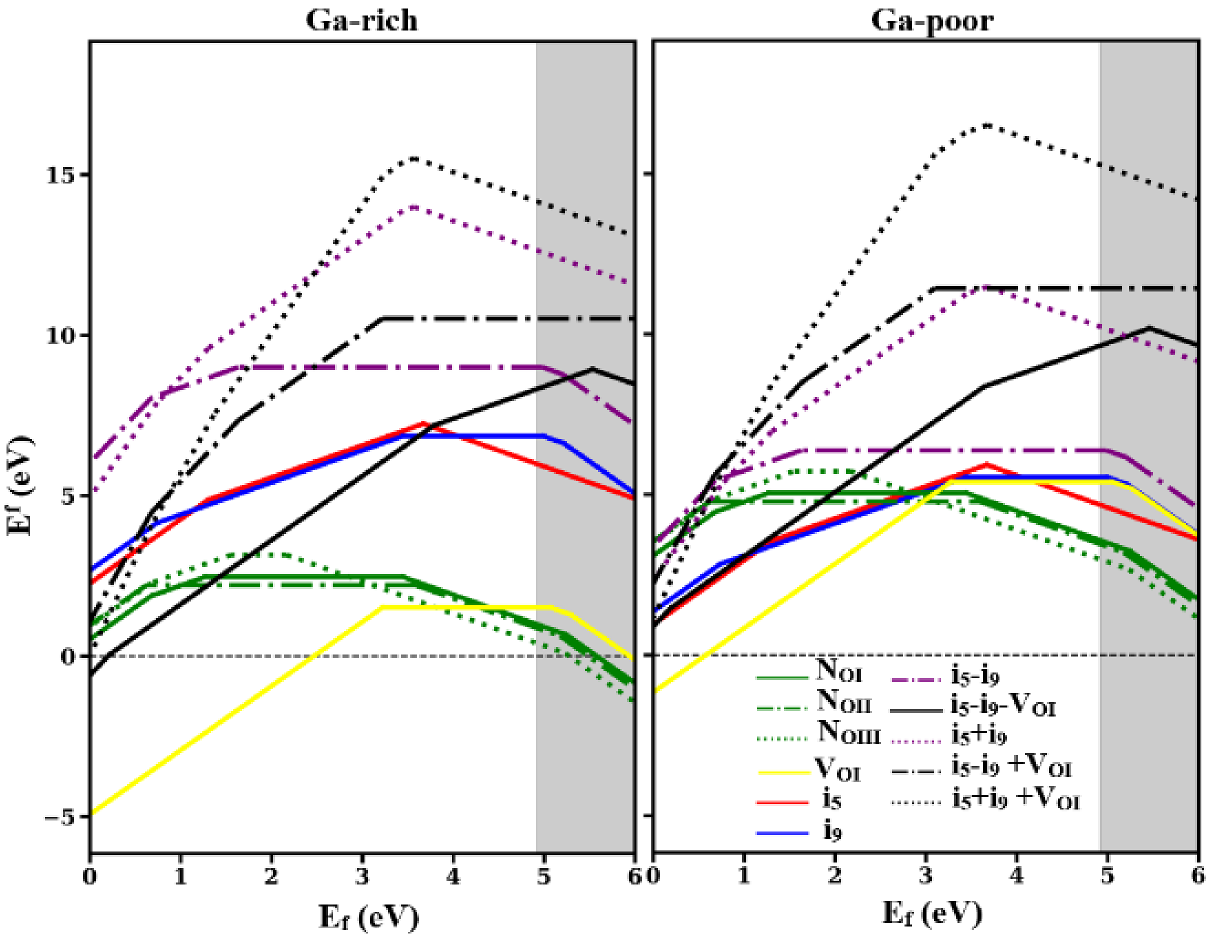

Figure S1. Formation energies of nitrogen-related defects in β-Ga_2O_3 as a function of the Fermi levels under Ga-rich and Ga-poor conditions. The considered configurations include substitutional N_O at inequivalent O sites (N_{OI}, N_{OII}, N_{OIII}), interstitial N at sites i5 and i9, their complexes (i5-i9 and i5-i9-V_{OI}), and the isolated oxygen vacancy V_{OI}. For comparison, the summed formation energies of isolated defects (i5+i9, i5-i9+V_{OI}, and i5+i9+V_{OI}) are also shown, representing the non-interacting limit where the defects are spatially separated. The lower formation energies of the interacting configurations indicate a thermodynamic preference for defect association. The Fermi level is referenced to valence band maximum.

S2. Raman Spectroscopy: Experimental Details and Results

Raman spectra were collected with a Horiba Jobin–Yvon Labram HR800 spectrometer equipped with an Olympus BX40 microscope with a 50× long-distance objective, a 600 groove/mm holographic grating, and a Peltier-cooled (1024 × 256 pixel) CCD detector. Excitation radiation was provided by a diode-pumped, frequency-doubled Nd:YAG laser (532 nm). The 520 cm^{-1} phonon mode from the silicon substrate was used for calibration.

In the work of Kajihara et al., a Raman band at approximately 2325 cm^{-1} was interpreted as molecular nitrogen interacting with the surrounding solid matrix, whereas a band near 2330 cm^{-1} was assigned to gaseous N_2 and was observed even in the absence of the sample.[9] An analogous spectral behavior is observed in the present Raman measurements, where a Raman feature shifted with respect to the gaseous N_2 band is detected (marked by an arrow in **Figure S2**). This observation is consistent with the formation of molecular-like nitrogen configurations in the implanted β-Ga_2O_3 layer.

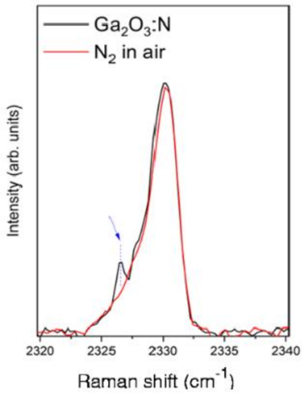

Figure S2. High-resolution Raman spectrum of the N-implanted β-Ga_2O_3 sample in the N_2 vibrational region. The arrow indicates a Raman feature shifted relative to the gaseous N_2 band. Similar spectral behavior was reported by Kajihara et al.[9], who assigned the shifted band to molecular nitrogen interacting with the surrounding solid matrix.

At the same time, Raman spectroscopy alone cannot unambiguously determine the local atomic environment of nitrogen. Therefore, the principal conclusions of the present work rely on the combination of bulk-sensitive N K-edge XANES measurements performed in the total fluorescence yield mode and FDMNES simulations, which directly probe the local electronic structure of nitrogen. The Raman results are intended as complementary evidence and provide independent experimental support for the interpretation derived from the XANES analysis.

References

[1] Kresse, G.; Hafner, J. Ab initio molecular-dynamics simulation of the liquid-metal–amorphous-semiconductor transition in germanium. *Phys. Rev. B*. **1994**, 49, 14251.

[2] Kresse, G.; Furthmüller, J. Efficiency of ab-initio total energy calculations for metals and semiconductors using a plane-wave basis set. *Comput. Mater. Sci.* **1996**, 6, 15.

[3] Kresse, G.; Furthmüller, J. Efficient iterative schemes for ab initio total-energy calculations using a plane-wave basis set. *Phys. Rev. B*. **1996**, 54, 11169.

[4] Kresse, G.; Joubert, D. From ultrasoft pseudopotentials to the projector augmented-wave method. *Phys. Rev. B*. **1999**, 59, 1758.

[5] Shokri, A.; Melikhov, Y.; Syryanyy, Y.; Demchenko, I.N. Point Defects in Silicon-Doped β-Ga_2O_3: Hybrid-DFT Calculations. *ACS Omega*. **2023**, 8, 43732.

[6] Heyd, J.; Scuseria, G. E.; Ernzerhof, M. Hybrid functionals based on a screened Coulomb potential. *J. Chem. Phys.* **2003**, 118, 8207.

[7] Peelaers, H.; Lyons, J.L.; Varley, J.B.; Van de Walle, C.G. Deep acceptors and their diffusion in Ga_2O_3, *APL Mater.* **2019**, 7, 022519.

[8] Passlack, M.; Hunt, N.E.J.; Schubert, E.F.; Zydzik, G.J.; Hong, M.; Mannaerts, J.P.; Opila, R.L.; Fischer, R.J. Dielectric properties of electron-beam deposited Ga_2O_3 films. *Appl. Phys. Lett.* **1994**, 64, 2715.

[9] Kajihara, K.; Hirano, M.; Takimoto, Y.; Skuja, L.; Hosono, H. Diffusion of nitrogen molecules in amorphous SiO_2. *Appl. Phys. Lett.* **2007**, *91*, 071904.
